# Indicator Development for the Evaluation of User‐Researcher Collaboration in Health Research: A Co‐Designed, Modified Delphi‐Study

**DOI:** 10.1111/hex.70880

**Published:** 2026-09-21

**Authors:** Lotte Verweij, Judith Safford, Saskia Oesch, Myrta Kohler, Thomas Zurbrügg, Tanja Brülhart, Marion Jourdan, Cristina De Biasio Marinello, Rahel Naef

**Affiliations:** ^1^ University of Zurich, Medical Faculty, Implementation Science in Nursing Zurich Switzerland; ^2^ University Hospital Zurich, Center for Clinical Nursing Science Zurich Switzerland; ^3^ Patient Research Partner, RheumaCura Foundation Bern Switzerland; ^4^ Patient and Family Member Research Partner Zurich Switzerland

**Keywords:** co‐design, health research, indicator, modified Delphi method, patient and public involvement and engagement, user‐researcher collaboration

## Abstract

**Introduction:**

Partnering with patients, close others and the public (users), termed patient and public involvement and engagement (PPIE), is essential to ensure relevant and impactful health research. To date, definitions of effective collaborations between users and researchers to plan and evaluate such collaborations are scarce. Therefore, building consensus on what makes user‐researcher collaborations effective and genuine is an important step. The goal of this co‐designed study was to define and establish expert agreement on key quality indicators for the evaluation of such partnerships in health research.

**Methods:**

We conducted a three‐round modified Delphi‐study (12/2023 – 07/2024). The first round included a participatory expert workshop with seven users and seven researchers, facilitated by two citizen science experts, to discuss and identify potential indicators, which were then refined by four user‐researcher delegates. In the second round, these indicators were assessed in a survey among the workshop participants for their relevance, clarity and completeness. In the final round, three user‐researcher delegates operationalized the redefined and reduced indicators, which were assessed for their relevance, clarity and ease of response in another survey with an extended group of 24 users and researchers.

**Results:**

The participatory expert workshop resulted in a set of 35 potential indicators. In the first survey (response rate 11/14), suggestions were made to optimise the indicator's clarity and completeness, and 5/35 indicators were considered (partially) redundant. Hence, indicators were merged, redefined, and formulated into 30 statements. In the second survey (response rate 13/24), indicators were evaluated as partly or fully relevant, with some suggestions to optimise clarity and make indicators easier to respond to (ease of response). The 30 quality indicators were subsequently categorised around structures (n = 9), processes (n = 9) and outcomes (n = 12) of PPIE.

**Conclusion:**

In this co‐developed, modified Delphi‐study, we brought user and researcher expertise together to identify and reach expert consensus on key indicators to evaluate user‐researcher collaboration in health research. This study resulted in an operationalized set of indicators relevant to both users and researchers using PPIE in health research. Next, the proposed indicators and their operationalization should be validated in practice.

**Patient or Public Contribution:**

This study was co‐designed and co‐conducted by the patient and family member advisory board and health researchers of the Family Support in Intensive Care Units (FICUS) trial. The patient and family member advisory board includes a patient expert (TZ) and three family member experts (TB, MJ, CdBM) with lived expertise in the field of critical illness or trauma, and is led by JS, who is an experienced patient research partner. Since this study's aim was to develop indicators for the evaluation of PPIE in health research from both users' and researchers' perspectives, collaboration in this study was a matter of course.

## 1 Introduction

The inclusion of patients, close others and the public, or ‘users', in research, also known as Patient and Public Involvement and Engagement (PPIE), is key to ensure meaningful and relevant health research to those it seeks to serve [1, 2, 3]. Despite increasing evidence that the use of PPIE in health research leads to more targeted health interventions and increased recruitment and retention rates, the integration of PPIE in research projects is often lacking [4, 5, 6, 7, 8]. Researchers remain sceptical about the usefulness and benefits of PPIE and are often reluctant to engage in it due to limited time and resources [5, 9, 10, 11]. Funding agencies and policymakers now increasingly require and support the integration of PPIE in health research projects, which creates incentives for research co‐production, but also increases the risk of tokenism. Caused by tokenism, meaning that PPIE is conducted only superficially or symbolically, participation may result in harm and has no meaningful impact on the research design or conduct [12, 13, 14, 15, 16, 17]. To achieve its potential for improvement, PPIE in research projects requires careful reflection and planning to avoid negative experiences for both users and researchers, which may arise for instance, due to power imbalances, role conflicts, disagreements about the project focus and outcomes, imbalanced time investment, or lack of or limited reimbursement [9, 15, 17, 18, 19, 20]. At the same time, the need for careful and comprehensive evaluation and learning from PPIE in health research has also been stressed [21, 22].

Recent advances to support researchers and users with structured integration of PPIE in health research and its evaluation have produced a series of PPIE logic models, guidance, practice and evaluation tools [21, 23, 24, 25]. For example [26]) published a European Patients' Academy on Therapeutic Innovation (EUPATI) guidance for PPIE, including suggested working practices, user involvement activities, and compensation‐ and written agreements [27]) proposed guidance for authentic and sustainable user‐researcher partnership, based on foundational principles such as respect, equity, and transparency. The Co‐Design Evaluation Framework by Peters and colleagues (2024) guides the evaluation of process and impact of research co‐design based on seven clusters such as the people within and outside the co‐design group, the co‐design context and research processes. The Center of Excellence on Partnership with Patients and the Public (CEPPP) has released a comprehensive guide that defines ‘best practices’ in planning, doing, and evaluating PPIE in research [28]. Most recently, the Patient‐Centred Outcomes Research Institute (PCORI) updated their framework, including 28 concepts organised in five domains to support instrument development for the evaluation of PPIE [29]. These guidelines provide important foundations for user‐researcher collaboration. They are useful to inform the development of instruments and tools to structure and evaluate the processes and outcomes of health research collaboration and engagement. However, they do not all offer practical or evaluation tools themselves.

Practical and evaluation tools to facilitate the process of collaboration and evaluation also exist. For instance the GRIPP2 reporting checklists by [30]) serves as a tool to ensure the quality of reporting of PPIE activity in research publications, which may also inform the planning, conduct and evaluation of user‐researcher collaboration. We also identified several specific instruments to evaluate user‐researcher collaboration and engagement. Examples are the Evaluating Public and Patient Involvement in Interventional Research Checklist (EPPIIC) reflecting three sub‐scale, i.e. policy and practice, participatory culture and influence and impact [24] and the Critical Outcomes of Research Engagement (CORE) components to assess the impact of user engagement in research which include criteria such as patient‐centeredness, meaningfulness, and team collaboration from user and researcher perspective [31]. Furthermore, the Patient and Public Engagement Evaluation Tool (PPEET) was developed, including four core principles of engagement: integrity, influence and impact, participatory culture, collaboration and common purpose for the evaluation from the user, project leader and leadership perspective [32, 33]. Finally, the 22‐items Patient Engagement in Research Scale (PEIRS) questionnaire evaluates meaningful user engagement and includes concepts as procedural requirements, convenience, and feeling valued for the evaluation from user perspective [34, 35].

These instruments have been thoroughly developed, but only the PEIRS‐22 questionnaire has been validated [34]. While the CORE, PPEET and EPPIIC assess engagement practices from both user and researcher perspectives, as recommended [36, 37], the PEIRS‐22 examines the quality of engagement from a user perspective only. In addition, these tools stem from Canada and the UK with sparse contributions from the European and German‐speaking regions. Hence, rigorous and co‐designed evaluation tools continue to be limited despite recent advances [7, 24, 38]. In addition, the need for comprehensive approaches to evaluate PPIE impact from various perspectives has been stressed [39]. To add to and expand the current body of knowledge on evaluating PPIE, we choose a participatory approach to define and develop expert consensus on indicators to evaluate user‐researcher collaboration in health research from both user and researcher perspectives.

## 2 Materials & Methods

### 2.1 Aim

This study aimed to build expert consensus on quality indicators for the evaluation of user‐researcher collaboration in health research, using a participatory approach.

### 2.2 Design

**Figure 1:**
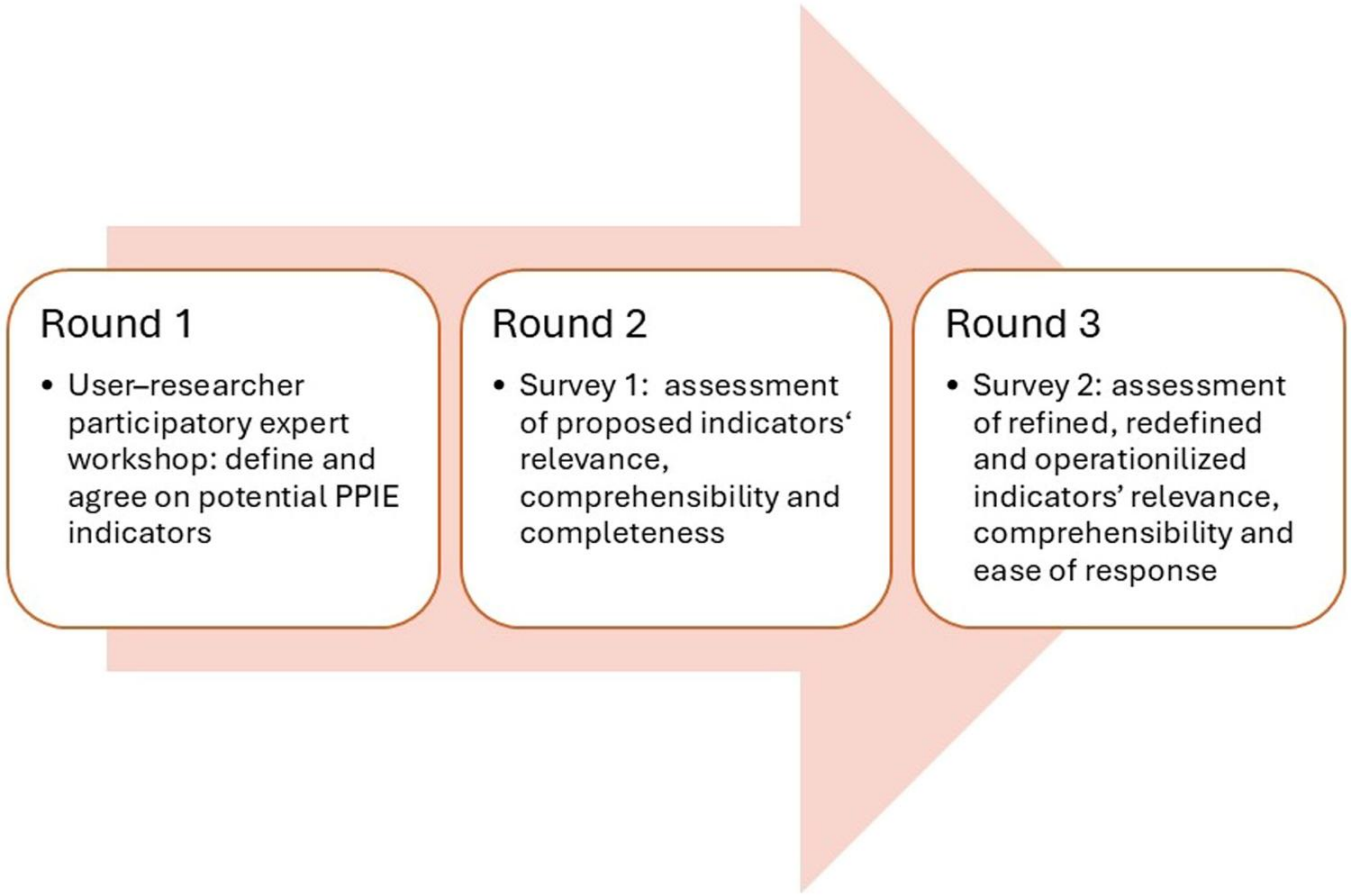
Delphi‐study process.

A three‐round modified Delphi‐study with PPIE users and researchers in the expert panel was performed between December 2023 and July 2024. Delphi methods are applied to develop consensus on a given topic by a group of experts, which is obtained through a multi‐staged study most often through surveys [40, 41]. Delphi studies have four key features, namely anonymity, iteration, controlled feedback and aggregation of group response [42]. These features can be applied in various formats. For instance, the process can be modified to include opportunities for open feedback or (expert) meetings so that participants can discuss their views [36] and which were relevant to the study process. Within the first round of our Delphi study (December 2023 – January 2024), a user‐researcher participatory expert workshop was held to openly discuss and identify potential indicators for PPIE in health research, see Figure 1. The participatory expert workshop was followed by a session with four user‐researcher delegates who clustered and defined the primary set of indicators. In the second round (February – April 2024), the indicators were evaluated for their relevance, clarity and completeness in an anonymous survey of the first‐round participants. This enabled responses and opinions without the influence of (unintended) hierarchical or other influencing factors. Based on the survey results, the indicators were refined and reduced. As part of round three (May – July 2024), three user‐researcher delegates operationalized the indicators into questions which were thereafter assessed for relevance, clarity and ease of response in a second anonymous survey by an extended group of user‐researcher experts.

The study is reported using the ACCORD (Accurate Consensus Reporting Document) Delphi reporting guidelines [43].

### 2.3 Framework

To structure and categorise the quality indicators for user‐researcher collaboration in health research, we applied the Donabedian framework, which is widely recognised for assessing healthcare quality [44, 45, 46]. The framework distinguishes three domains: structure, process, and outcome. Structure refers to the organisational and contextual attributes of the setting, including resources, personnel, infrastructure, and policies. Process encompasses the activities and interactions between participants, emphasising how engagement occurs. Outcome relates to the effects of PPIE on health research, including its impact on research quality, relevance, and participant experiences. This framework provides a systematic approach to cluster PPIE quality indicators.

### 2.4 Context and Participants

Eight users and eight researchers with PPIE experiences in health research in Switzerland were recruited to participate in the expert panel. Inclusion criteria included German language knowledge and PPIE experience as either a patient or family research partner (user), or as a health researcher. Three users and one researcher were engaged in an ongoing research collaboration, in the Family Support in Intensive Care Units (FICUS) trial [47, 48]. Other users and researchers were recruited through Swiss organisational bodies known for their focus on PPIE in research (EUPATI Switzerland; a patient group known as the Swiss PPIE Network; Swiss National Science Foundation) or were known to the authors for their PPIE expertise and experience in research projects, representing both clinical and academic settings. We actively contacted recommended individuals by e‐mail and followed up by telephone. If individuals showed interest in participation, they received written study information including the participation requirements and expectations and options for reimbursement of travel expenses and payment for their invested time. Approximately 2 weeks prior to the workshop participants received an informed consent form which was collected prior to the workshop.

For Delphi round three, ten additional user‐ and researcher experts (five each) were recruited through these same means to enable an outsider perspective.

### 2.5 Data Collection

#### 2.5.1 Delphi Round 1 – Participatory Expert Workshops

In December 2023, a 4.5‐h participatory expert workshop was organised to discuss and identify characteristics of (un)successful PPIE, which served as a basis for the quality indicator development. The workshop was held at the University campus, which was easy to travel to and barrier free. Two citizen science experts (specialised in scientific research with citizen scientists, i.e. involving members of the general public) facilitated the workshop. In total, seven users and seven researchers participated. The participatory expert workshop was based on the Most Significant Change Technique in which storytelling has a central role [49]. In this approach, each participant contributes a personal story, describing the most significant experiences with PPIE. This approach enables an equal, balanced dialogue between people with different backgrounds and experiences on a certain topic [49].

To prepare for the workshop, all participants were asked to write down one story on a PPIE experience, which had left a lasting impression on them. The story needed to include a reflective aspect, which explains the impact of their experience on their role or expertise as user or researcher, such as positive or negative feelings, thoughts, expectations, added value gained, improvements or deteriorations. The workshop began with an introductory round of all participants, their relation to and experience with PPIE and a declaration of conflicts of interest, if applicable. The ground rules were clarified in advance and included confidentiality, equality and respectful communication. Thereafter, the workshop aim was presented and the Most Significant Change Technique was applied [49].

#### 2.5.2 Delphi Round Two – Survey

The second Delphi round included an anonymous semi‐structured online survey among users and researchers from the first round to rate their agreement on the indicators' *relevance* to evaluate PPIE practices (Visual Analogue Scale = VAS, 1‐9), *clarity* of the indicators (3‐point Likert‐scale: yes, partly, not), and *completeness* of the set of indicators to evaluate PPIE practices (yes/no). Additionally, open text fields were offered to add suggestions for the indicators or to motivate the assessment. At the end of the survey, we asked for the following individual characteristics: age, sex, role in PPIE (user or researcher), years of experiences with PPIE and an open text field to elaborate on participated PPIE activities. Respondents received the survey in February 2024 and were asked to complete the survey within 6 weeks. A reminder email was sent after 3 weeks.

#### 2.5.3 Delphi Round Three – Indicator Refinement and Second Survey

In the third Delphi round, one user (JS) and two researchers (LV, SO) delegates refined and reduced the indicators set based on the results from round two. If potential indicators were rated either with a median VAS < 4 or assessed as not or partly *relevant*, *clear*, or as *incomplete*, the suggestions from the open text field were carefully considered for adjustment or omission of the indicator. Afterwards, indicators were operationalized into questions and discussed by three user‐researcher delegates before implementing them in a second online survey. An extended user‐researcher group (n = 24) was invited to rate the operationalized indicators' *relevance* to evaluate PPIE practices, the indicators' *clarity* and *ease of response* by a 3‐point Likert‐scale (yes, partly, not) in a second anonymized survey. For each question, there was the option to comment in an open text field. If the indicators were rated as either not or partly *relevant, clear* or *easy to respond to*, the suggestions from the open text field were again carefully considered, and the operationalized indicators further refined. Respondents received the survey in June 2024 and were asked to complete the survey within 6 weeks. A reminder email was sent after 3 weeks.

### 2.6 Data Analysis

For rounds two and three, data were collected in and exported from UNIPARK, an online platform used to implement surveys [50]. Descriptive statistics were performed by calculating absolute numbers and percentages, and medians with ranges in R Version 4.3.1 and in Microsoft Excel 365.

### 2.7 Ethics

This study was submitted to the Institutional Review Board of the Faculty of Medicine, University of Zurich (MeF‐Ethik‐2023‐02), which evaluated and waived the need for ethical approval for the research project. Participants received a study information package and signed a written informed consent form prior to the workshop. Completing the surveys in round two and three was considered as permission for the use of the data.

### 2.8 Definition of Key Terms

**Table:** 

Key term	Definition
Patient and public involvement and engagement (PPIE)	“Patient and Public Involvement and Engagement (PPIE) is understood as the active inclusion of patients, close others (e.g., family members or carers), and members of the public (‘users’) as partners in determining research priorities, designing studies, conducting research, and disseminating findings, with research being carried out *with* or *by* them rather than *to*, *about* or *for* them.” Involve patients | NIHR
Indicators	A quality indicator is a measurable element that helps assess how well a process, service, or product meets defined standards or expectations [44].
Delphi study	Delphi methods are applied to develop consensus on a given topic by a group of experts, which is obtained through a multi‐staged study most often through surveys [40, 41]

## 3 Results

### 3.1 Delphi Round One – Participatory Expert Workshop

**Figure 2:**
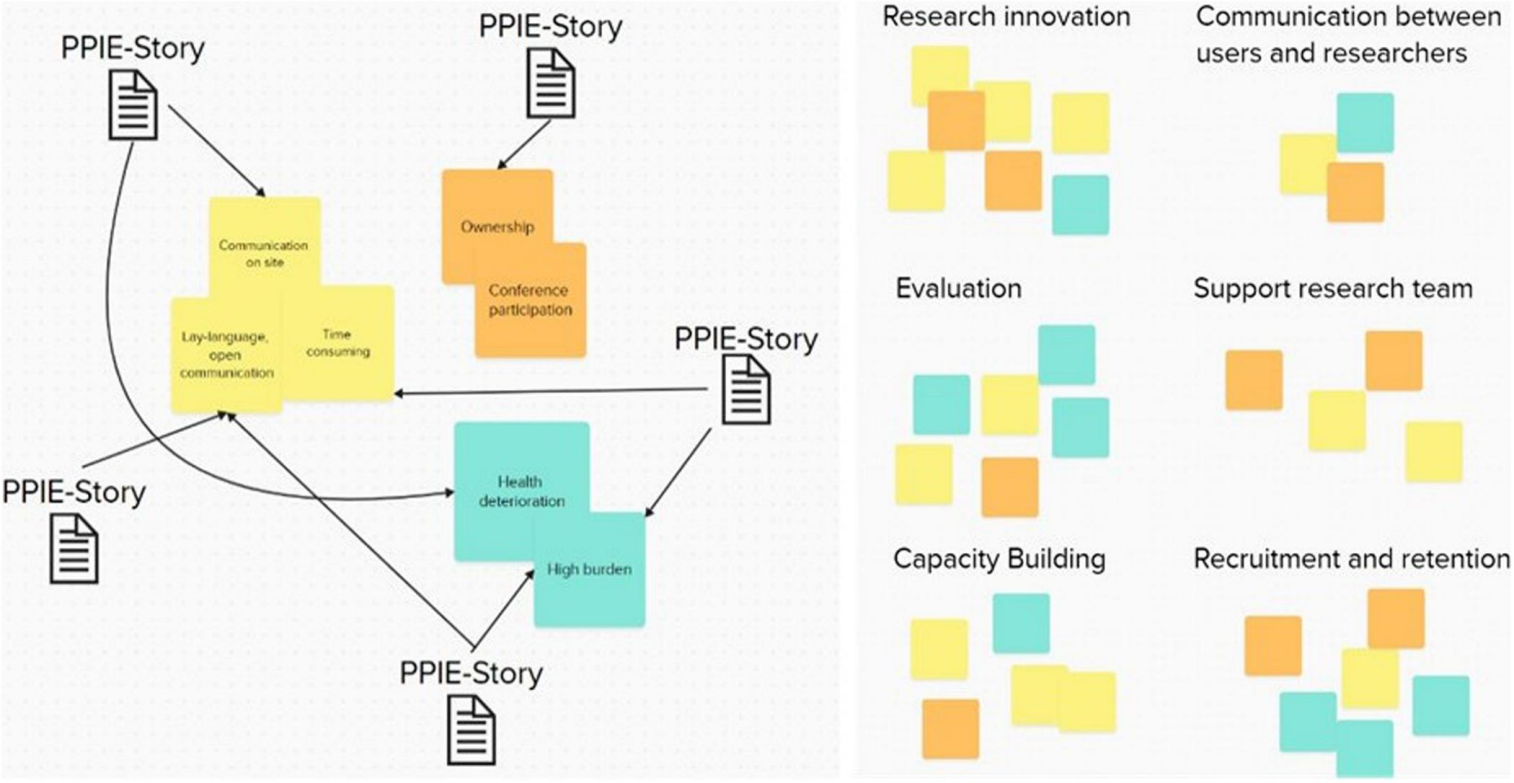
Processing story content. Left side: personal PPIE story content was written down on post‐its. Right side: forming clusters out of the PPIE story content.

Of 16 invited persons, seven users and seven researchers attended the expert workshop. Two experts could not attend due to personal circumstances. The workshop included several phases. First, participants presented their story to share their experiences in four small groups of three to four persons with the same role (user or researcher). This was followed by a round of reflection, first individually and thereafter in pairs. Notes on the content of the stories were written down on post‐its. After the exchange, the post‐its were clustered by common themes, see Figure 2. Second, the four small groups were merged into two mixed groups of users and researchers. Representatives of each small group presented the main findings to the other group members. The clusters were discussed between the users and researchers with respect to their relevance, and differences and commonalities were sought. Overarching themes were formulated for each evolving post‐it cluster, based on a consensus approach. Then, the moderators asked the groups to select a few example themes to work through and to formulate indicators. Questions such as “what personal PPIE stories does the theme contain?”, “what were the concrete activities and circumstances of the stories that formed the themes?”, and “what were the moments of success that makes the described stories a relevant PPIE story?” were discussed and formed the basis to develop the expert group consent‐based indicators. The last part of the participatory expert workshop was done in plenum. Each group moderator presented a few exemplary indicators and the way they were formulated. At the end, the participants were briefed about the next steps, and a reflection round was made to evaluate the procedure.

The workshop was then followed by a 4‐h user‐researcher delegates workshop to further indicate and formulate indicators in the same way as in the participatory expert workshop. Participants were one user (JS) and two researcher (LV and SO) delegates and one citizen science facilitator to maintain continuity. The delegates further developed a set of indicators that captured all themes/characteristics of user‐researchers collaboration generated during the workshop. In total, 35 potential indicators were indicated and clustered into structure, process, and outcome indicators, according to Donabedian [44], see Table 1. The clustering of indicators enabled to differentiate between indicators on structures for PPIE practice, indicators on PPIE working processes and indicators on impact of PPIE activities on research and involved users and researchers.

**Table 1:** PPIE indicator development through three Delphi rounds^a^.

	Round 1. Indicators as worded after the participatory expert workshop	Round 2. Updated indicators after the first survey	Round 3. Finalised and operationalized indicators after the second survey^b^
**Structure indicators**			
1	*Requirements for users and researchers*	*Requirements for users and researchers*	
	The requirements for users and researchers are defined in advance (e.g. training and experience, availability, personal requirements such as courage, motivation).	The requirements for users and researchers are defined in advance (e.g. training and experience, availability, skills).	The personal requirements necessary for the research project (e.g. training and experience, availability, skills) were defined in advance.
2	*Description of roles and/or tasks*	*Description of roles and/or tasks*	
	Role and/or task descriptions are available for all users and researchers (e.g. management or co‐management, creation or co‐creation, consultation, feedback).	Role and/or task descriptions are available for all users and researchers (e.g. management or co‐management, creation or co‐creation, consultation, feedback).	Role and/or task descriptions are available for all users and researchers (e.g. management or co‐management, creation or co‐creation, consultation, feedback).
3	*Agreements*	*Agreements*	
	Agreements are in place for all users and researchers.	Written agreement for the research project between users and researchers was reached at an early stage.	Written agreements for the collaboration were made at an early stage.
4	*Clarification of duration and effort*	*Duration and effort*	
	The expected duration and the expected effort of the engagement are defined.	The expected duration and effort of the participation of users and researchers is defined.	The expected duration and effort of my involvement in the project have been discussed with me.
5	*Financial compensation forms*	*Financial compensation forms*	
	Possible forms of compensation have been agreed (e.g. financial compensation for cooperation, compensation for travel expenses, etc.).	All receive the offer of appropriate financial compensation (e.g. expenses, salary, vouchers).	The possible compensation for my cooperation (e.g. fees, travel expenses, vouchers) has been discussed and agreed with me.
6	*Training opportunities*	*Training opportunities*	
	Training opportunities relevant to the project have been discussed.	Training opportunities relevant to the project and PPIE have been discussed.	Relevant training opportunities for the project on PPIE were discussed with me.
7	*Dissemination opportunities*	*Dissemination opportunities*	
	Dissemination opportunities (e.g. authorship) are defined in advance.	Issues of co‐authorship, acknowledgement in publications and the type of recognition in other forms of dissemination are clarified in advance with users and researchers concerned.	Issues relating to co‐authorship, citation in publications and the type of recognition in other forms of dissemination were clarified with me in advance.
8	*Accessible infrastructure*	*Infrastructure*	
	An accessible infrastructure is guaranteed for all users and researchers and/or appropriate alternative participation options are offered (virtual participation).	An accessible infrastructure is guaranteed for all users and researchers and/or appropriate alternative participation options are offered (virtual participation).	During the research project, an accessible infrastructure was guaranteed for me at all times and/or appropriate alternative ways of participating in meetings were offered (e.g. virtual participation).
9	*Access to information*	*Digital information access*	
	Digital access to information and documents is guaranteed for all users and researchers.	Digital access to necessary information and documents is guaranteed for all users and researchers.	During the research project, I had digital access to the necessary information and documents at all times.
**Process indicators**			
10	*Getting to know each other*	*Getting to know each other & clarifying roles*	
	All users and researchers involved have the opportunity to get to know each other as team members.	All users and researchers involved have the opportunity to get to know each other as team members and clarify roles in the research project.	I had ample opportunity to get to know all users and researchers involved and to clarify who had which role within the research project.
11	*Individual expectations*	*Clarification of expectations*	
	All users and researchers have the opportunity to share their individual assumptions and expectations regarding the PPI collaboration with the team.	All users and researchers have the opportunity to share their individual assumptions and expectations regarding the PPI collaboration with the team.	I had ample opportunity to share my personal assumptions and expectations regarding collaboration with users and researchers involved.
12	*Team collaboration rules*	*Team collaboration rules*	
	Rules for team collaboration have been discussed and defined (e.g. feedback, respectful interaction, confidentiality, everyone is an expert, dealing with conflicts.	The rules for collaboration between users and researchers are jointly discussed and defined (e.g. feedback, respectful interaction, equality, confidentiality, dealing with conflicts).	Rules for collaboration between users and researchers (e.g. feedback, respectful interaction, equality, confidentiality, dealing with conflicts) have been jointly discussed and defined.
13	*Defined communication processes*	*Communication processes*	
	Communication processes within the team are defined (when, how and with whom do you communicate?)	Communication processes between users and researchers are defined (when and how to communicate and with whom).	Communication processes between users and researchers were clearly defined.
14	*Equal participation*	*Participation*	
	It is ensured that users and researchers can have their say and meetings are not dominated by individual voices.	During meetings, it is ensured that users and researchers have equal say.	Users and researchers involved were given equal opportunities to speak at meetings.
15	*Impact of participation in the research project*	*Impact of participation in the research project*	
	Research coordinators sensitise all users and researchers about individual experiences and what participation in the research project can trigger in each individual.	Research coordinators sensitise all users and researchers about individual experiences and what participation in the research project can trigger in each individual.	I was made aware by the research coordinators of possible effects that could be triggered by my personal experiences in relation to the research project.
16	*Finding solutions for collaboration challenges*	*Finding solutions for collaboration challenges*	
	Research coordinators create space for all users and researchers to share (invisible) hurdles and barriers to teamwork.	Research coordinators create space for all users and researchers to address hurdles and barriers to cooperation and to jointly find a solution.	Research coordinators have created sufficient space to address hurdles and barriers to cooperation between users and researchers and to jointly find solutions.
17	*Team collaboration evaluation*	*Team collaboration evaluation*	
	The team collaboration (users and researchers) is regularly evaluated and adapted if necessary.	The collaboration between users and researchers is regularly evaluated and adapted as necessary.	The collaboration between users and researchers was regularly evaluated and adapted as necessary.
18	*Comprehensible communication*	*Tailored communication*	
	Communication (written and verbal) is understandable and appropriate for the recipient.	In all forms of communication (written/oral), communication is understandable and appropriate to the recipient (meetings, conferences, workshops, individual discussions, documents and papers).	Written and verbal communication (e.g. meetings, conferences, workshops, individual discussions, documents and papers) was comprehensible and appropriate to the recipient at all times.
**Outcome indicators**			
19	*Appreciation within the project team*	*Appreciation within the project team*	
	The expertise of users/researchers is valued within the project team.	Users and researchers experience mutual interest and are valued for their different expertise.	I was valued as a person with my expertise by all the users and researchers involved.
20	*Mutual respect*	*Mutual respect*	
	Users/researchers feel that their individual needs, emotions and vulnerability are respected within the research team.	Users and researchers feel that their individual needs, emotions and vulnerability are respected within the research team.	I felt respected in my individual needs, emotions and vulnerability by the users and researchers at all times.
21	*Task‐oriented participation*	*Task‐oriented participation*	
	Users/researchers are equally involved in the ongoing project planning, decision‐making and implementation (e.g. inclusion in scheduling, deadlines).	Users and researchers are involved in the ongoing research project according to their role/task (e.g. inclusion in scheduling, deadlines, decision‐making processes).	I have been involved in the ongoing research project according to my role/task (e.g. involvement in scheduling, deadlines, decision‐making processes).
22	*Opportunity for personal/professional development*	*Opportunity for personal and/or professional development*	
	Users/researchers have the opportunity to continuously develop personally and professionally (e.g. in the form of further training).	Users and researchers have the opportunity to develop personally and/or professionally.	Working on the project gave me the opportunity to develop personally and/or professionally.
23	*Recognition/confirmation of collaboration*	*Confirmation of collaboration*	
	Users/researchers receive appropriate recognition for their work (e.g. through proof, certificates, diplomas, references).	Users and researchers receive appropriate confirmation for their work (e.g. through proof, certificates, diplomas, references).	I have received appropriate confirmation of my work on the research project (e.g. through proof, certificates, documents, testimonials, references).
24	*Successful implementation*	*Successful implementation*	
	PPIE application is visible through successful implementation processes in organisations.	Goal‐oriented cooperation between users and researchers contributes to the successful implementation of research results in healthcare institutions.	I am convinced that research results can be implemented more successfully in healthcare institutions through effective collaboration between users and researchers.
25	*Relevant research questions*	*Relevant research questions and research*	
	The PPIE application is visible through the joint pursuit of research objectives relevant to the public/population/target group.	A goal‐oriented cooperation between users and researchers leads to the pursuit of research goals that are relevant to the public/target group.	I am convinced that meaningful cooperation between users and researchers will lead to the pursuit of research goals that are relevant to the public/target group.
26	*Scientific results*	*Scientific results*	
	PPIE application is visible through scientific results (e.g. publications, media, events, associations) that are understandable to the public/population/target group.	The goal‐oriented cooperation between users and researchers is made visible through scientific results that are understandable and freely available to the public/target group in the form of publications, media contributions, events, etc.	I am convinced that cooperation between users and researchers will lead to comprehensible and accessible study results in the form of publications, media articles, events, etc. for the public/target group.
27	*Public interest and research*	*Public interest and research*	
	PPIE application is visible through increased interest and credibility of PPI in research and the public (e.g. through successful dissemination of study results).	The goal‐oriented cooperation between users and researchers is visible through an increased interest in and increased credibility of PPIE in the research community and the public.	I am convinced that close cooperation between users and researchers will lead to increased interest in and increased credibility of PPIE in the research community and among the public.
28	*Network building*	*Network building*	
	PPIE application is visible through extended network building (e.g. research and target group).	The goal‐oriented cooperation between users and researchers is visible through the expansion of their own network.	I am convinced that the cooperation of all those involved in research will lead to the formation of an extended network, for example between researchers and their target population.
29	*Successful recruitment processes*	*Successful recruitment processes*	
	PPIE application is visible through the successful recruitment of study participants in the research project.	The goal‐oriented cooperation between users and researchers is visible in the successful recruitment of study participants in the research project.	I am convinced that active cooperation between users and researchers will lead to the successful recruitment of study participants for the research project.
30	*Retention of study participants*	*Retention of study participants*	
	The use of PPIE is visible through the retention of study participants in the research project (preventing early study drop‐out).	The goal‐oriented cooperation between users and researchers is visible in the retention of study participants in the research project (prevention of early drop‐out).	I am convinced that through the active cooperation of all people involved in the research, study participants can be kept in the research project and premature dropouts can be prevented.
**Indicators integrated and omitted after round 1**			
	*Role clarification*		
	Roles are clarified together with all users and the researchers.	Integrated into indicator 10	
	*Financial compensation*		
	Users/researchers receive appropriate monetary compensation (e.g. expenses, salaries)	Integrated into indicator 5	
	Equal participation		
	Users/researchers are equally involved in e.g. the ongoing decision‐making processes.	Integrated into indicator 14	
	*Equal collaboration*		
	Users/researchers are treated equally within the project team.	Integrated into indicator 14 and 19	
	*Mutual interest*		
	Users/researchers experience mutual authentic interest.	integrated into indicator 20	

Abbreviation: PPIE, patient and public involvement and engagement

^a^ Please inform the corresponding author when using the indicators for evaluation purposes. When reporting the indicators, this reference should be cited.

^b^ Answer categories for the operationalized indicators on PPIE for survey application: 0. not applicable, 1. strongly disagree, 2. disagree, 3. agree, 4. strongly agree.

### 3.2 Delphi Round Two – Survey

The 35 potential indicators were assessed by 11 of the 14 (78.6%) invited participants, including five researchers and six users. Reasons for non‐response are unknown. Most were female (82%) and about half of them were between 50 and 59 years old (46%), followed by 27% being between 30 and 39 years old, 18% between 40 and 49 years, and 9% were 60 years or older. Their median PPIE experience was 5 years (range 0.5 – 30) and examples of PPIE activities they were involved in were participation in and leading health research including activities, participation in (inter)national conferences on PPIE, and participation in and leading courses on PPIE.

**Figure 3:**
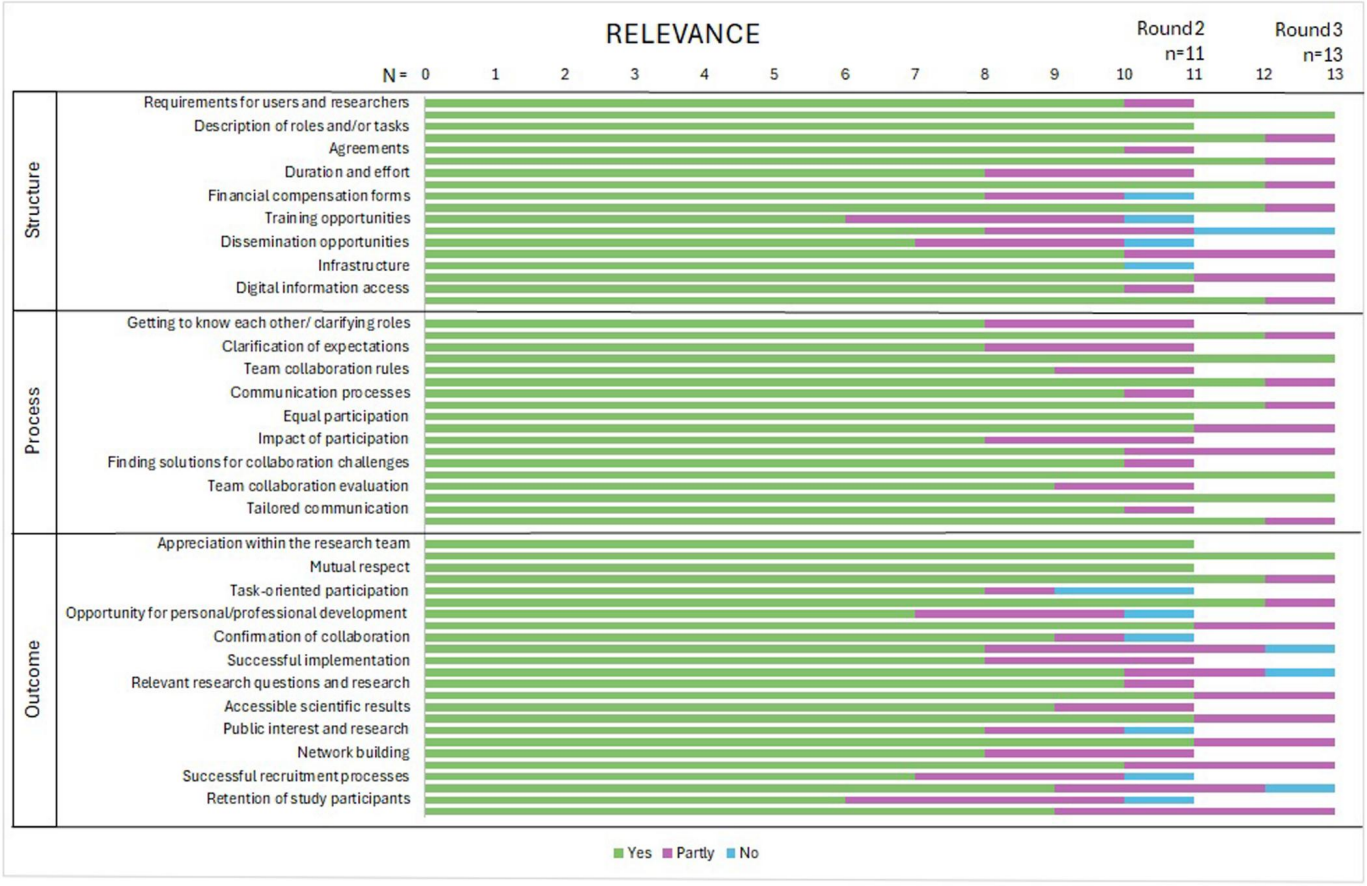
Delphi round two and three ‐ relevance of PPIE indicators. Each indicator contains two rows. The top row for each item reflects the round 2 survey on indicators' relevance assessed by n = 11 respondents. The second row for each item reflects the round 3 survey on the revised indicators' relevance, assessed by n = 13 respondents.

**Figure 4:**
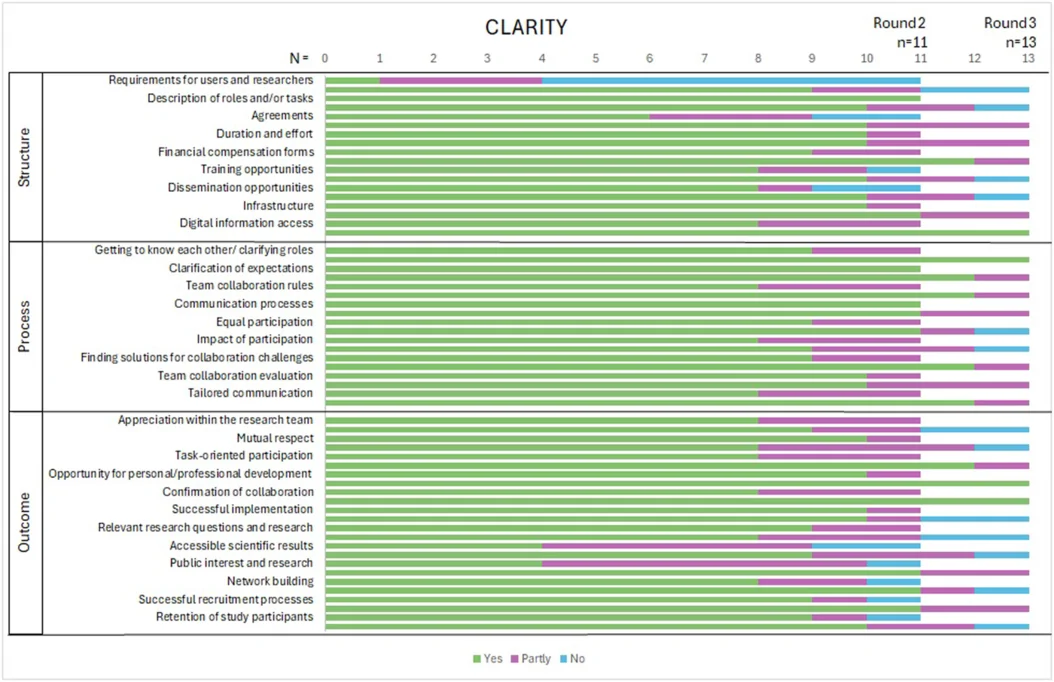
Round two and three ‐ clarity of PPIE indicators Each indicator contains two rows. The top row for each item reflects the round 2 survey on the indicators' clarity, assessed by n = 11 respondents. The second row for each item reflects the round 3 survey on the revised indicators' clarity, assessed by n = 13 respondents.

According to the predefined cut‐off of a median VAS ≥ 4, all proposed indicators were considered *relevant*, see Table 2. According to the three answer categories *yes, partly or not*, 25 indicators were assessed as *partly* and 10 as *not relevant* by some participants, see Figure 3. In relation to clarity, 27 indicators were assessed as *partly* and 9 as *not clear* by some participants, see Figure 4. Suggestions for optimisation or changes in the open text fields included terminology, redundancy and applicability of indicators for both users and researchers. Additional suggestions were made in the open text field to supplement the proposed indicators. For example, it was suggested that it is important to reflect the aim of conducting more significant and relevant research from the perspective of the target population in an indicator, and that compensation is key and closely linked to treating people as equals. There are many ways to reward user involvement, including offering professional development opportunities. It is important to highlight the various options and build in flexibility in the indicator on agreement because participation and commitment can vary over time. Based on the results and the suggestions from the open text fields, a delegation of the research team (JS, LV and SO) reduced the initial set of 35 potential indicators by merging redundant indicators. In addition, definitions were refined, which resulted in a set of 30 indicators (nine structure, nine process, 12 outcome), see Table 1. Examples of merged/refined indicators are ‘role clarification’ which was integrated into indicator 10 ‘getting to know each other & clarifying roles’, and the indicator ‘mutual interest’ which was integrated and combined in indicator 20 ‘mutual respect’, see Table 1.

**Table 2:** Delphi round two – survey – relevance of PPIE indicators.

Nr.	Indicator	VAS* 1–9 median (min‐max)
**Structure**		
1	Requirements for users and researchers	8 (4‐9)
2	Description of roles and/or tasks	8 (7‐9)
3	Agreements	8 (5‐9)
4	Clarification of duration and effort	8 (5‐9)
5	Financial compensation forms	9 (6‐9)
6	Training opportunities	7 (3‐8)
7	Dissemination opportunities	7 (1‐9)
8	Accessible infrastructure	8 (1‐9)
9	Access to information	8 (5‐9)
**Process**		
10	Getting to know each other	9 (6‐9)
11	Individual expectations	9 (5‐9)
12	Team collaboration rules	7 (5‐9)
13	Defined communication processes	8 (5‐9)
14	Equal participation	9 (7‐9)
15	Impact of participation in the research project	7 (5‐9)
16	Finding solutions for collaboration challenges	8 (5‐9)
17	Team collaboration evaluation	9 (5‐9)
18	Comprehensible communication	8 (5‐9)
**Outcome**		
19	Appreciation within the project team	8 (5‐9)
20	Mutual respect	9 (7‐9)
21	Task‐oriented participation	8 (5‐9)
22	Opportunity for personal/professional development	7 (3‐8)
23	Recognition/confirmation of collaboration	7 (1‐9)
24	Successful implementation	7 (4‐9)
25	Relevant research questions	9 (5‐9)
26	Scientific results	8 (5‐9)
27	Public interest and research	8 (3‐9)
28	Network building	7 (4‐9)
29	Successful recruitment processes	7 (1‐9)
30	Retention of study participants	7 (1‐9)
**Merged and redefined after round 2 (see** Table 1 **)**		
31	Role clarification	9 (5‐9)
32	Offer of financial compensation	8 (5‐9)
33	Task‐oriented participation project planning/implementation	8 (2‐9)
34	Equal participation	9 (6‐9)
35	Mutual interest	9 (6‐9)

* Survey round two indicators' relevance measured on a VAS: Visual Analogue Scale

### 3.3 Delphi‐Round Three – Survey

In total, 13 out of 24 (54%) invited user‐ and researcher experts participated in the second survey, of whom five researchers and eight users. Most participants were female (85%) and 53% were aged between 30 and 39 years, 31% between 50 and 59 years and 16% between 40 and 49 years. In total, 9/13 (69%) had already participated in the first and second Delphi‐rounds. Reasons for non‐response are unknown.

**Figure 5:**
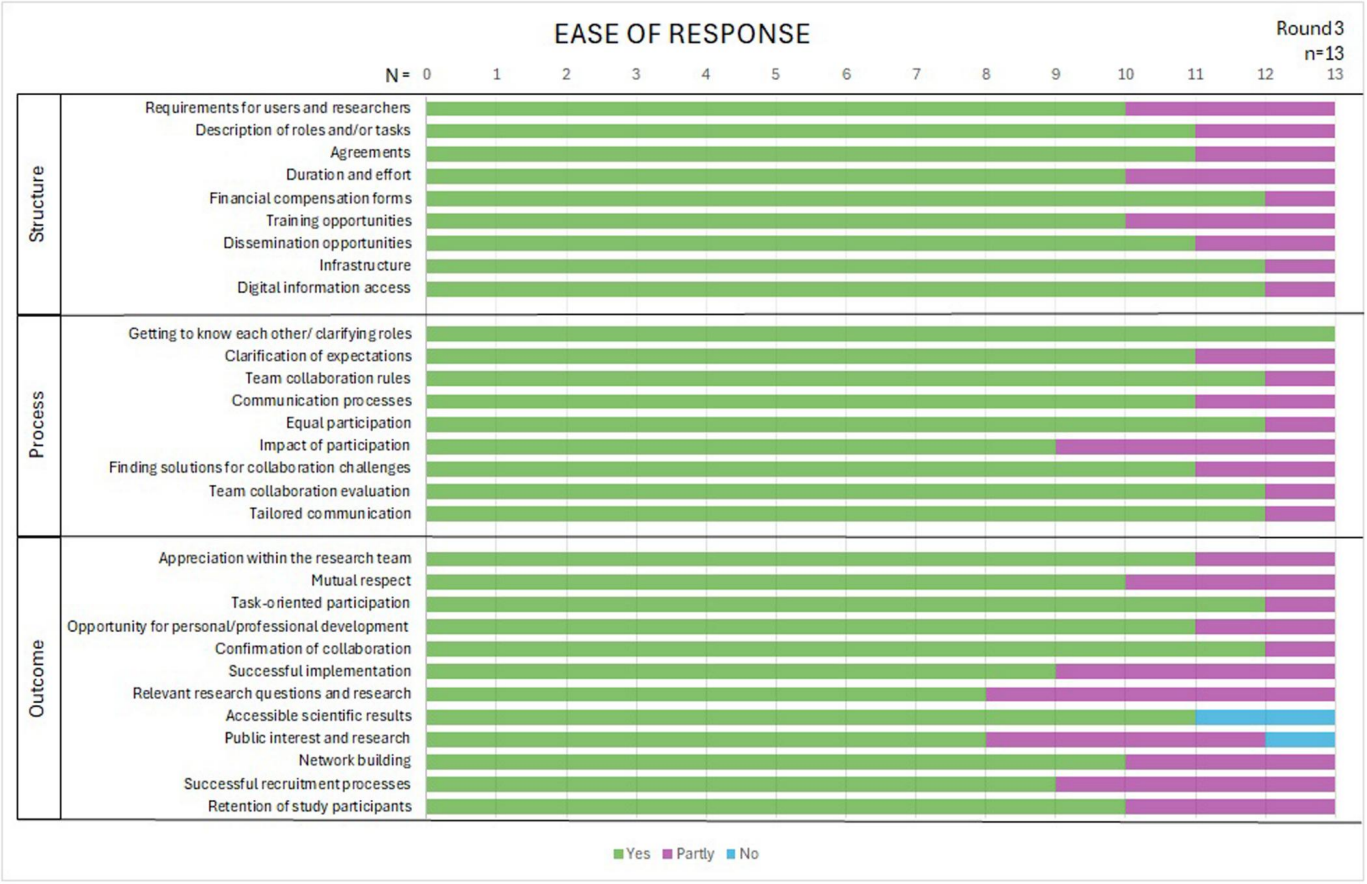
Round three ‐ ease of response to operationalized PPIE indicators. The operationalized indicators' ease of response is only evaluated in the third round and is assessed by n = 13 respondents.

Results show that proposed operationalized indicators were considered *relevant* (see Figure 3), *clear* (see Figure 4) and *easy to respond to* (see Figure 5) by most participants. According to the three answer categories *yes, partly* or *not*, 25/30 indicators were assessed as either *partly* (n = 25) or *not* (n = 4) *relevant*, 26/30 *partly* (n = 26) or *not* (n = 13) *clear* and 29/30 *partly* (n = 29) or *not* (n = 2) *easy to respond to* by some participants. Suggestions for optimisation or changes were made in open text fields. For instance, the relevance of the project phase was considered, as well as the provision of examples to explain certain terminology, such as ‘infrastructure', and the simplification of indicators. Based on these suggestions, 15 indicators (see Table 1: 1‐5, 7, 11‐15, 18, 9, 26, 28, 30) were refined and finalised within the same set of 30 indicators (i.e. 9 structure, 9 process, 12 outcome) by delegates of the research team (JS, LV and SO). No indicator needed to be omitted. Table 1 shows the process of refinement and reformulation of the indicators through from round one to three, Supporting file 1 provides the final (operationalized) indicator set.

## 4 Discussion

In this three‐round modified Delphi‐study we defined and built an expert consensus on relevant indicators for the evaluation of PPIE in health research incorporating the perspective of both users and researchers. The co‐designed and participative study process led to 30 operationalized indicators, comprising of nine structure‐, nine process‐ and 12 outcome indicators. The indicators may serve prospectively to guide PPIE processes in the preparation phase of research projects, to monitor the integration of PPIE activities and practices in ongoing research projects, and retrospectively to systematically and comprehensively evaluate PPIE in health research projects.

Our indicators are rooted in lived expertise of user‐researcher collaboration, which included both difficult and positive experiences. As such, they integrate user and researcher perspectives in a core set of quality indicators. In line with our expectations, the inductively generated and refined indicators have some conceptual overlap with other instruments [24, 31, 33, 34]. For example, properties such as communication and support, sharing experiences and opinions within the PPEET‐surveys [32], understandability and team collaboration within the CORE components [31], procedural requirements and team environment and interaction within the PEIRS‐22 survey [34], and properties such as a participatory culture and the influence and impact of PPIE activities within the EPPIIC checklists [24]. Despite these commonalties, which were particularly strong with the PEIRS‐22 survey [34], our indicators aim to evaluate collaboration from both user and researcher perspectives with the same tool, which contrasts with the PEIRS‐22, which assesses the user perspective alone. When aiming at equal collaborations in research projects, evaluation of user‐researcher collaboration should include all members of the research team involved in and affected by the collaboration activities. Quality indicators should be of relevance and applicable to both roles to ensure equality. Existing instruments focusing on the evaluation from both user and researcher perspective, such as the PPEET surveys [32, 33], the CORE components [31], or the EPPIIC checklists [24] have not been validated to date. Furthermore, existing frameworks focusing on users and researchers such as the Co‐design Evaluation Framework [38] and the Patient‐Centred Outcomes Research Institute [29], lack operationalized measures and include examples and suggestions only. In addition, our indicators cover the full spectrum from structures and processes to outcomes of user‐researcher collaboration. Rather than focusing primarily on the experience or impact of engagement, the indicators may therefore support a more comprehensive assessment of the conditions under which collaboration takes place, how collaboration is enacted, and what it achieved. Moreover, their intended use across the preparation, conduct and retrospective evaluation of PPIE in health research may provide a practical advantage by allowing research teams to identify areas for improvement not only after, but also during the collaboration.

The engagement of users as important stakeholders in health research is considered as one of the six core elements in the United Kingdom's Medical Research Council guidance for developing and evaluating complex health interventions and should be considered early and continually throughout the research process [51]. Despite the advantages, engaging users in research comes with certain risks [39], and has led to negative experiences among users as described by Richards and colleagues (2023). First, tokenism and involving users as a check mark in research projects is considered as an important pitfall in user engagement. Second, unconscious bias from researchers towards users, defined by the Canadian Institutes of Health Research (2024) as an ‘implicit, unintentional attitude or assumption that affects the way you think and act’, and by Fitzgerald and Hurst (2017) as ‘implicit bias’, poses a risk to user‐researcher collaborations because of the risk of introducing disparities. Third, the lack of physical and other support to users so they may fully participate as team members is mentioned as an additional risk, which includes, among other aspects, practical issues such as the timing of scheduled meetings, reimbursement of expenses and invested time, and being offered patient research partner training. Fourth, another pitfall is failing to recognise the vulnerability of users. Others have stressed the need to understand PPIE as a social practice that may not only result in benefit but could lead to disempowerment and harm if not conducted with caution [39]. PPIE may also be seen as a ‘conversation that supports mutual learning between researchers and the public' [52]. Our indicators may help overcome pitfalls and reductionist view on PPIE across research phases and enable it to be a reflected social practice or learning conversation. For example, our indicator set considers aspects of equality and shared power through indicators such as written agreements or having equal say during meetings. Aspects of support are addressed by indicators such as access to information and accessible infrastructures. Acknowledgement is demonstrated by indicators on financial compensation and dissemination opportunities and avoiding unconscious bias is addressed by indicators relating to pre‐defined rules for team collaboration and role clarification.

### 4.1 Study Strengths and Limitations

This study used a participative approach; it was co‐designed and co‐conducted in collaboration with users and researchers in both the research team and as participants, to represent both views throughout the entire study process. The modified Delphi technique allowed us to combine both open in‐depth discussions and anonymized responses in the agreement process. Even though unintended hierarchy, power differentials, and varying knowledge levels and expertise with PPIE may have been present, we found the open discussion during the participatory expert workshop essential to the development of quality indicators. The workshop enabled all participants to share thoughts, including experiences from different perspectives and to find commonalities among users and researchers through an in‐depth consent approach. To limit the influence of unintended hierarchy and of power differences, we chose to work with the Most Significant Change Technique that allowed all participants to give an equal workshop contribution by telling their own ‘story'. Still, there is a reasonable chance that some participants perceived hierarchical structures in the group, which may have influenced their level of contribution by feeling hindered in for instance their openness, honesty and criticism.

Despite our efforts, some study limitations should be acknowledged. The on‐site participatory expert workshop resulted in a relatively small user‐researcher expert group, which may not fully reflect the broader population of PPIE experts. Nevertheless, we deliberately initiated the Delphi study with a participatory expert workshop that drew on participants' individual PPIE experiences to identify common characteristics of successful and unsuccessful PPIE. This approach was chosen instead of a more traditional approach in which the characteristics assessed in the Delphi study would have been derived primarily from the existing literature. This method allowed for an open perspective on themes emerging from personal opinions and experiences, in contrast to more conventional methods. As a result, we may have overlooked themes identified in the literature that were not raised through this process. However, our indicators find an overlap with existing instruments confirming the relevance of the indicator content. Finally, the indicators were developed in German and translated for the purpose of this paper. A back‐ and forward cross‐cultural translation and adaptation procedure is yet to be done.

### 4.2 Future Perspectives

The current study represents the first step in developing indicators of user‐researcher collaboration quality from both user and researcher perspectives, covering the full spectrum of PPIE, including structures, processes and outcomes of user‐researcher collaboration. As a next step, the operationalized indicators were applied and tested to evaluate user‐researcher collaboration within the FICUS trial. The co‐designed FICUS trial evaluated the effect of a novel family nurse role in ICUs on family satisfaction with ICU care, family‐clinician communication, and cognitive and emotional support, compared with usual care [48, 53]. The FICUS trial incorporated an active patient and family member advisory board. Finally, the operationalized indicators need to proceed into a validation phase and need to be psychometrically tested in different research environments and cultures. Future validation will also offer an opportunity to demonstrate the added value of these indicators relative to key existing frameworks and evaluation instruments.

## 5 Conclusion

In this co‐developed, co‐conducted modified Delphi study, we developed expert consensus on quality indicators for the evaluation of user‐researcher collaboration in health research, and to do so, brought user and researcher perspectives, expertise, and priorities together, giving every voice equal say. The study resulted in 30 quality indicators, applicable from both user and research perspectives, on the structures, processes and outcomes of PPIE in health research projects. The indicator set can be used during study preparation, its conduct, and afterwards to evaluate the application of PPIE in health research projects across different fields, including clinical, community‐based or public health contexts. This study constitutes a first step in PPIE indicator development, contributing to an evolving body of research on measuring PPIE in health research. As such, the study may guide PPIE design and evaluation, extending a still modest toolbox to evaluate patient and public engagement in research. As a next step, our indicator set will need to be developed into a psychometrically validated measure.

## Author Contributions

**Lotte Verweij:** conceptualisation, funding acquisition, writing – original draft, investigation, methodology, validation, visualisation, writing – review and editing, software, formal analysis, project administration, data curation. **Judith Safford:** conceptualisation, funding acquisition, writing – review and editing, formal analysis, validation. **Saskia Oesch:** writing – review and editing, investigation, formal analysis, validation. **Myrta Kohler:** investigation, writing – review and editing, formal analysis, validation. **Thomas Zurbrügg:** funding acquisition, writing – review and editing, validation. **Tanja Brülhart:** funding acquisition, validation, writing – review and editing. **Marion Jourdan:** funding acquisition, writing – review and editing, validation. **Cristina De Biasio Marinello:** funding acquisition, validation, writing – review and editing. **Rahel Naef:** validation, conceptualisation, funding acquisition, writing – review and editing, methodology, supervision, resources.

## Ethics Statement

This study was submitted to the Institutional Review Board of the Faculty of Medicine, University of Zurich (MeF‐Ethik‐2023‐02), which evaluated and approved the research project.

## Conflicts of Interest

The authors declare no conflicts of interest.

## Supporting information


Supporting File

## Data Availability

Data are available upon reasonable request with the corresponding author.
