# Supplementary material for: Indicator Development for the Evaluation of User‐Researcher Collaboration in Health Research: A Co‐Designed, Modified Delphi‐Study
Source: Health Expect. 2026 Sep 21;29(5):e70880. doi: 10.1111/hex.70880 (PMC13591234; doi:10.1111/hex.70880)
Supplement: Supplementary file 1 [file HEX-29-e70880-s001.docx]

**Supplementary file**

**Patient and public involvement and engagement (PPIE) Indicators and Operationalized Statements^1^**

Verweij, L., Safford, J., Oesch, S., Kohler, M., Zurbrügg, T., Brühlhart, T., Jourdan, M., De Biasio Marinello, C., & Naef, R. (2026). Indicator development for the evaluation of user-researcher collaboration in health research: A co-designed, modified Delphi-study *Health Expectations*.

| **Nr.** | **Title Indicator** | **Indicator** | **Operationalized statements**  **Instruction:** Please indicate to what extent you agree with the following statements *(1. strongly disagree, 2. disagree, 3. agree, 4. strongly agree, 0= not applicable).* |
| --- | --- | --- | --- |
| **Structure indicators** | | | |
| **1** | **Requirements for users and researchers** | The requirements for users and researchers are defined in advance (e.g. training and experience, availability, skills). | The personal requirements necessary for the research project (e.g. training and experience, availability, skills) were defined in advance. |
| **2** | **Description of roles and/or tasks** | Role and/or task descriptions are available for all users and researchers (e.g. management or co-management, creation or co-creation, consultation, feedback). | Role and/or task descriptions are available for all users and researchers (e.g. management or co-management, creation or co-creation, consultation, feedback). |
| **3** | **Agreements** | Written agreement for the research project between users and researchers was reached at an early stage. | Written agreements for the collaboration were made at an early stage. |
| **4** | **Duration and effort** | The expected duration and effort of the participation of users and researchers is defined. | The expected duration and effort of my involvement in the project have been discussed with me. |
| **5** | **Financial compensation forms** | All receive the offer of appropriate financial compensation (e.g. expenses, salary, vouchers). | The possible compensation for my cooperation (e.g. fees, travel expenses, vouchers) has been discussed and agreed with me. |
| **6** | **Training opportunities** | Training opportunities relevant to the project and PPI have been discussed. | Relevant training opportunities for the project on PPI were discussed with me. |
| **7** | **Dissemination opportunities** | Issues of co-authorship, acknowledgement in publications and the type of recognition in other forms of dissemination are clarified in advance with users and researchers concerned. | Issues relating to co-authorship, citation in publications and the type of recognition in other forms of dissemination were clarified with me in advance. |
| **8** | **Infrastructure** | An accessible infrastructure is guaranteed for all users and researchers and/or appropriate alternative participation options are offered (virtual participation). | During the research project, an accessible infrastructure was guaranteed for me at all times and/or appropriate alternative ways of participating in meetings were offered (e.g. virtual participation). |
| **9** | **Digital information access** | Digital access to necessary information and documents is guaranteed for all users and researchers. | During the research project, I had digital access to the necessary information and documents at all times. |
| **Process indicators** | | | |
| **10** | **Getting to know each other/ clarifying roles** | All users and researchers involved have the opportunity to get to know each other as team members and clarify roles in the research project. | I had ample opportunity to get to know all users and researchers involved and to clarify who had which role within the research project. |
| **11** | **Clarification of expectations** | All users and researchers have the opportunity to share their individual assumptions and expectations regarding the PPI collaboration with the team. | I had ample opportunity to share my personal assumptions and expectations regarding collaboration with users and researchers involved. |
| **12** | **Team collaboration rules** | The rules for collaboration between users and researchers are jointly discussed and defined (e.g. feedback, respectful interaction, equality, confidentiality, dealing with conflicts). | Rules for collaboration between users and researchers (e.g. feedback, respectful interaction, equality, confidentiality, dealing with conflicts) have been jointly discussed and defined. |
| **13** | **Communication processes** | Communication processes between users and researchers are defined (when and how to communicate and with whom). | Communication processes between all users and researchers were clearly defined. |
| **14** | **Participation** | During meetings, it is ensured that users and researchers have equal say. | Users and researchers involved were given equal opportunities to speak at meetings. |
| **15** | **Impact of participation in the research project** | Research coordinators sensitize all users and researchers about individual experiences and what participation in the research project can trigger in each individual. | I was made aware by the research coordinators of possible effects that could be triggered by my personal experiences in relation to the research project. |
| **16** | **Finding solutions for collaboration challenges** | Research coordinators create space for all users and researchers to address hurdles and barriers to cooperation and to jointly find a solution. | Research coordinators have created sufficient space to address hurdles and barriers to cooperation between users and researchers and to jointly find solutions. |
| **17** | **Team collaboration evaluation** | Collaboration between users and researchers is regularly evaluated and adapted as necessary. | Collaboration between users and researchers was regularly evaluated and adapted as necessary. |
| **18** | **Tailored communication** | In all forms of communication (written/oral), communication is understandable and appropriate to the recipient (meetings, conferences, workshops, individual discussions, documents and papers). | Written and verbal communication (e.g. meetings, conferences, workshops, individual discussions, documents and papers) was comprehensible and appropriate to the recipient at all times. |
| **Outcome indicators** | | | |
| **19** | **Appreciation within the project team** | Users and researchers experience mutual interest and are valued for their different expertise. | I was valued as a person with my expertise by all the users and researchers involved. |
| **20** | **Mutual respect** | Users and researchers feel that their individual needs, emotions and vulnerability are respected within the research team. | I felt respected in my individual needs, emotions and vulnerability by the users and researchers at all times. |
| **21** | **Task-oriented participation** | Users and researchers are involved in the ongoing research project according to their role/ task (e.g. inclusion in scheduling, deadlines, decision-making processes). | I have been involved in the ongoing research project according to my role/task (e.g. involvement in scheduling, deadlines, decision-making processes). |
| **22** | **Opportunity for personal/professional development** | Users and researchers have the opportunity to develop personally and/or professionally. | Working on the project gave me the opportunity to develop personally and/or professionally. |
| **23** | **Confirmation of collaboration** | Users and researchers receive appropriate confirmation for their work (e.g. through proof, certificates, diplomas, references). | I have received appropriate confirmation of my work on the research project (e.g. through proof, certificates, documents, testimonials, references). |
| **24** | **Successful implementation** | Goal-oriented cooperation between users and researchers contributes to the successful implementation of research results in healthcare institutions. | I am convinced that research results can be implemented more successfully in healthcare institutions through (effective) collaboration between users and researchers. |
| **25** | **Relevant research questions and research** | A goal-oriented cooperation between users and researchers leads to the pursuit of research goals that are relevant to the public/ target group. | I am convinced that (meaningful) cooperation between users and researchers will lead to the pursuit of research goals that are relevant to the public/ target group. |
| **26** | **Scientific results** | The goal-oriented cooperation between users and researchers is made visible through scientific results that are understandable and freely available to the public/target group in the form of publications, media contributions, events, etc. | I am convinced that cooperation between users and researchers will lead to comprehensible and accessible study results in the form of publications, media articles, events, etc. for the public/target group. |
| **27** | **Public interest and research** | The goal-oriented cooperation between users and researchers is visible through an increased interest in and increased credibility of PPIE in the research community and the public. | I am convinced that (close) cooperation between users and researchers will lead to increased interest in and increased credibility of PPIE in the research community and among the public. |
| **28** | **Network building** | The goal-oriented cooperation between users and researchers is visible through the expansion of their own network. | I am convinced that the cooperation of all those involved in research will lead to the formation of an extended network, for example between researchers and their target population. |
| **29** | **Successful recruitment processes** | The goal-oriented cooperation between users and researchers is visible in the successful recruitment of study participants in the research project. | I am convinced that (active) cooperation between users and researchers will lead to the successful recruitment of study participants for the research project. |
| **30** | **Retention of study participants** | The goal-oriented cooperation between users and researchers is visible in the retention of student participants in the research project (prevention of early drop-out). | I am convinced that through the (active) cooperation of all people involved in the research, study participants can be kept in the research project and premature dropouts can be prevented. |

Abbreviation: PPIE=Patient and Public Involvement and Engagement

^1^Please inform the corresponding author when using the indicators for evaluation purposes. When reporting the indicators, this reference should be cited.
